# Transmission of Cryphonectria Hypovirus 1 (CHV1) to *Cryphonectria radicalis* and In Vitro and In Vivo Testing of Its Potential for Use as Biocontrol Against *C. parasitica*

**DOI:** 10.3390/ijms252212023

**Published:** 2024-11-08

**Authors:** Pedro Romon-Ochoa, Pankajini Samal, Jelena Kranjec Orlović, Alex Lewis, Caroline Gorton, Ana Pérez-Sierra, Mick Biddle, Lisa Ward

**Affiliations:** 1 Forest Research, Plant Pathology Department, Alice Holt Lodge, Wrecclesham GU104LH, Surrey, UK; pankajini.samal@forestresearch.gov.uk (P.S.); mick.biddle@forestresearch.gov.uk (M.B.); lisa.ward@forestresearch.gov.uk (L.W.); 2 Institute of Forest Protection and Wildlife Management, University of Zagreb Faculty of Forestry and Wood Technology, Svetošimunska Cesta 23, 10000 Zagreb, Croatia; jkranjec@sumfak.unizg.hr; 3 Forest Research, Tree Health Diagnostics and Advisory Service (THDAS), Alice Holt Lodge, Wrecclesham GU104LH, Surrey, UK; alex.lewis@forestresearch.gov.uk (A.L.); caroline.gorton@forestresearch.gov.uk (C.G.); ana.perez-sierra@forestresearch.gov.uk (A.P.-S.)

**Keywords:** biocontrol, *Cryphonectria parasitica*, *Cryphonectria radicalis*, hypovirus 1 (CHV1), transmissions, virulence

## Abstract

Cryphonectria hypovirus 1 (CHV1) is successful in controlling *Cryphonectria parasitica*, the causal agent of chestnut blight, but little is known regarding its transmission to other fungi, for example the European *Cryphonectria radicalis*. In this study, CHV1 was transmitted (circa 200,000–800,000 copies/microliter) to seven *C. radicalis* isolates from infected *C. parasitica*. Reverse transmission to virus-free *C. parasitica* (European 74 testers collection) was achieved, although it was less successful (250–55,000 copies/µL) and was dependent on the vegetative compatibility (VC) group. In *C. radicalis*, the virus infection led to colony colour change from pink to white and smaller colonies, dependent on the virus concentration. The virus was concentrated in the colony edges, and vertically transmitted to 77% of conidia. However, several in vitro experiments demonstrated that *C. radicalis* was always outcompeted by the blight fungus, only suppressing the pathogen between its 25–50% inoculum level. It presented good secondary capture only when acting as a pioneer. Two types of *in planta* assays (individual and challenge inoculations) were undertaken. *Cryphonectria radicalis* behaved as a saprotroph, while chestnut blight fungus behaved as an aggressive pathogen, and lesions after treatment with *C. radicalis* were no smaller in general, only when using cut branches. Overall, the results showed that infected *C. radicalis* was unable to control cankers.

## 1. Introduction

Chestnut blight caused by the fungus *Cryphonectria parasitica* (Murrill) M. E. Barr. was first introduced from Asia into North America in the late nineteenth century, with a first outbreak in the New York Zoo, killing over 3.5 billion American chestnut trees (*Castanea dentata* (Marsh.) Borkh.) in 50 years [1], almost leading to the extinction of this highly susceptible tree species. In Europe, it was first observed on the susceptible sweet chestnut (*Castanea sativa* Mill.) in Italy in 1938 [2], and since then, the pathogen has been introduced multiple times to most continental European countries [3] and England [4,5,6,7,8,9,10]. Symptoms include crown dieback, rapid-growing cankers on the trunk and/or branches, epicormic growth below cankers, orange fungal sporulation erupting through swollen lenticels, and often whitish, pale mycelial fans beneath the bark.

Cryphonectria hypovirus 1 (CHV1) is a type of virus belonging to the Hypoviridae family [11,12]. Hypoviruses are RNA viruses located in the cytoplasm membrane vesicles of their fungal hosts, without a coat protein, which replication form is dsRNA [13] and their main mode of transmission to other isolates/fungi occurs horizontally via hyphal anastomosis and vertically via conidia. Cryphonectria hypovirus 1 acts as a biocontrol agent of chestnut blight in Europe and some parts of North America (Virginia, Wisconsin, Maryland), and it is released there because it causes reduced *C. parasitica* growth, pigmentation, sporulation, and virulence [14].

In England, CHV1 was detected for the first time in November 2017 [6] and since then, low levels of the presence and concentration of this virus have been recorded in the environment. Some isolates collected in England have been subsequently infected by a high concentration of CHV1 through transmissions [8,9] from highly infected *C. parasitica* isolates from continental Europe of the same/most proximate VCG. As a result of these transmissions, Forest Research now hold a bank of hypovirulent isolates suitable for deployment in field trials on infected sites. However, their deployment in England is subject to regulatory consent, which is still to be received, due to *C. parasitica* being a regulated quarantine pest (even in its attenuated form when infected with the hypovirus). The complicated regulatory position, and the general high diversity of the vegetative compatibility types of *C. parasitica* in England [6], are likely to make the effective biocontrol of *C. parasitica* in Britain challenging. Therefore, there is a need for a more comprehensive knowledge of other potential biocontrol agents which could potentially serve as alternative ‘vehicles’ for the virus, to aid effective management of the disease.

Considering this, a few *Cryphonectria radicalis* (Schw. ex Fries) Barr. [15] isolates were found to be naturally infected with the virus in a previous study in England [7]. The tasks of the present study were: (i) transmit the mycovirus during horizontal transmission assays into seven isolates of *C. radicalis*, the closely related saprotrophic (or very mild pathogen) native (European lineage) species; (ii) investigate the impacts of this mycovirus infection on *C. radicalis* fungal phenotype; and (iii) determine both in vitro and in planta the competitive interactions between the fungus *C. parasitica* and the infected and non-infected *C. radicalis* isolates.

## 2. Results

### 2.1. Isolates

Most of the fungal cultures obtained from lesions, during Forestry Commission surveillance for *C. parasitica*, represented isolates of *C. parasitica* (511 isolates). However, the non-pathogenic native species, *C. radicalis*, was confirmed as well (29 isolates). These twenty-nine *C. radicalis* isolates are indicated in Table 1, some of which were preserved and used in this study.

### 2.2. Transmissions

CHV1 was initially transmitted from *C. parasitica* isolate WAR706, gained by past transmissions [8], into *C. radicalis* isolate ABB154 (which previously tested negative for the virus) with a final concentration of 333,833.11 viral copies per microliter (Table 2). Absence of traces of the *C. parasitica* donor in the final *C. radicalis* colony, subcultured from the most distal part of the recipient culture, was proven by negative results from dual hydrolysis real-time PCR for *C. parasitica* [16], while the 1:10 to 1:10,000 positive controls amplified well.

From the infected isolate ABB154, *C. radicalis*, the virus was transmitted to other six un-infected *C. radicalis* cultures with final concentrations ranging between circa 200,000 to nearly 800,000 viral copies per microliter (Table 3).

When crossing back to a collection of *C. parasitica* VCGs (EU1 to EU74), each highly infected *C. radicalis* culture transmitted the virus predominantly to only one VCG indicated in Table 4. Virus loads differed with the most successful transmission from FRA135 to EU62 (final concentration circa 55,000 copies/µL). The least successful was from BOS158 to EU9 (only 250 copies/µL).

We detected that EU16 harbors itself the mycovirus because it was positive among all crosses, and because it was positive after obtaining a new EU16 plug from −80 °C which was positive for the CHV1 virus following the same molecular procedure and timings explained in material and methods.

### 2.3. Colony Colour, Area, Colony Parts, Conidiation and Conidia Transmission

**Figure 1:**
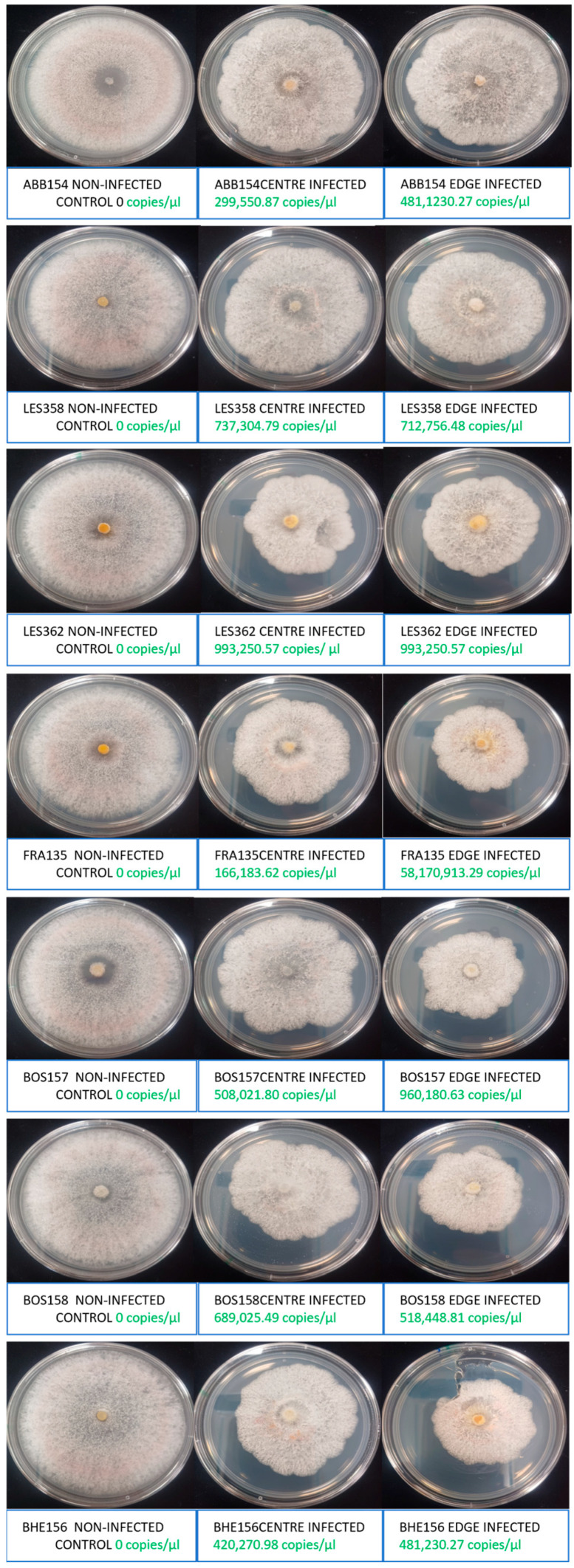
Pictures of 7 days old cultures of the seven CHV1 mycovirus infected *C. radicalis* isolates (subbed from the centre or the edge) and the respective non-infected isogenic cultures, with indication of the copy number of the virus.

The CHV1 mycovirus infection of seven *C. radicalis* isolates led to colony colour change from pink to white, passing through an intermediate stage between colours (Figure 1). Cycle threshold (Ct) value negatively correlated with colony colour (Pearson correlation coefficient −0.855, *p* < 0.0001) (Table 5). As Ct values increased, the binary notation system decreased (0 pink, 1 intermediate, 2 white). The loss of colony colour (pink pigment) was also negatively correlated with colony growth area. White colonies (without the pigment) expressed the smallest growth (Pearson correlation coefficient −0.737, *p* < 0.0001).

Ct value also negatively correlated with colony type (0, non-infected control, 1 subbed from the centre, 2 subbed from the edge) meaning that the virus was more concentrated in the youngest parts of the colony at the edge than in the centre (Pearson correlation coefficient −0.830, *p* < 0.0001) (Table 5). Convergently, with lower Ct values, the colonies were smaller in area (Pearson correlation coefficient 0.843, *p* < 0.0001) (Table 5).

CHV1 infection did not affect *C. radicalis* sporulation rate in a significant manner (Pearson correlation coefficient 0.374, *p* = 0.095) (Table 5).

However, the virus was present in *C. radicalis* conidia. Indeed around 77% of the single spore cultures of *C. radicalis* obtained harboured the virus with a relatively high viral concentration (Table 6). The isolates that provided the most vertical transmission in this way were LES358 and LES362, while BHE156 only transmitted the virus to 40% of its conidia.

### 2.4. Competition

**Figure 2:**
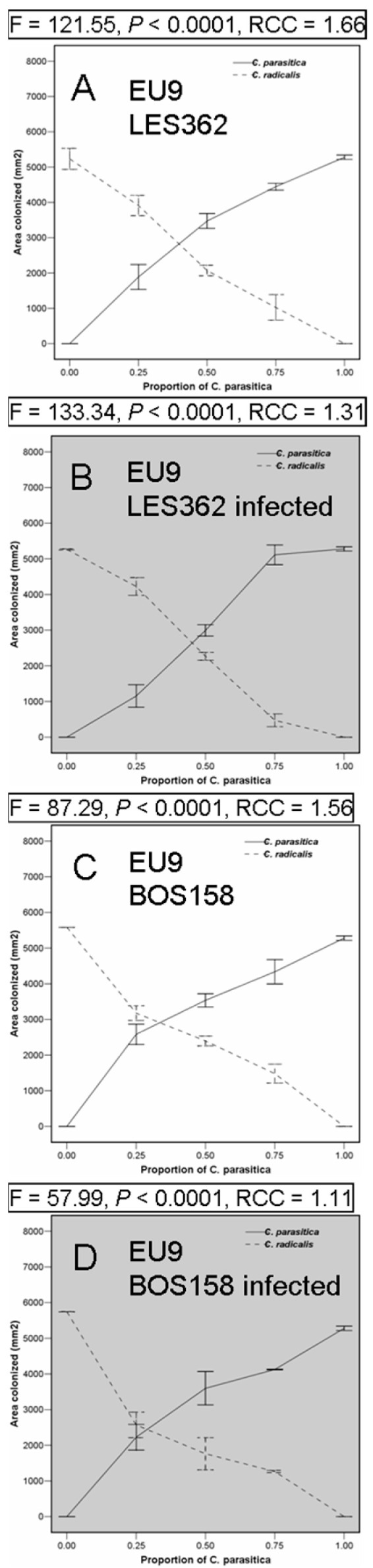
Differential competition of chestnut blight fungus, *Cryphonectria parasitica* EU9 vegetative compatibility group, interactions with *Cryphonectria radicalis* isolates LES362 and BOS158, non-infected or infected (highlighted in grey) by the CHV1 mycovirus. The significance of the decrease of *C. parasitica* growth areas as its proportion is lower and indicated by the *p*-value. Relative Crowding Coefficient, when over one, *C. parasitica* is outcompeting *C. radicalis*. See Section 4.4 for experimental details.

Overall, at the end of the whole experiment, *Cryphonectria parasitica* always outcompeted *C. radicalis* (Relative Crowding Coefficients > 1) (Figure 2). The RCC was always greater when using uninfected controls of *C. radicalis*. The fact that, during this trial, LES362 contained the highest concentration of CHV1 and thus would be slower growing, may explain why *C. parasitica* outcompeted this isolate slightly better than the less-infected BOS158.

*Cryphonectria radicalis* outcompeted the pathogen at the more favourable pathogen ratios of 50% and below (LES362), and 25% and below (BOS158). The standard error bars where the lines intersect (Figure 2) were always shorter when using infected *C. radicalis*, which might be explained by a degree of virus transmission occurring, especially when using BOS158 which has earlier been shown to be compatible with the competing *C. parasitica* culture (WAR706, VCG EU9).

### 2.5. Primary Capture

**Figure 3:**
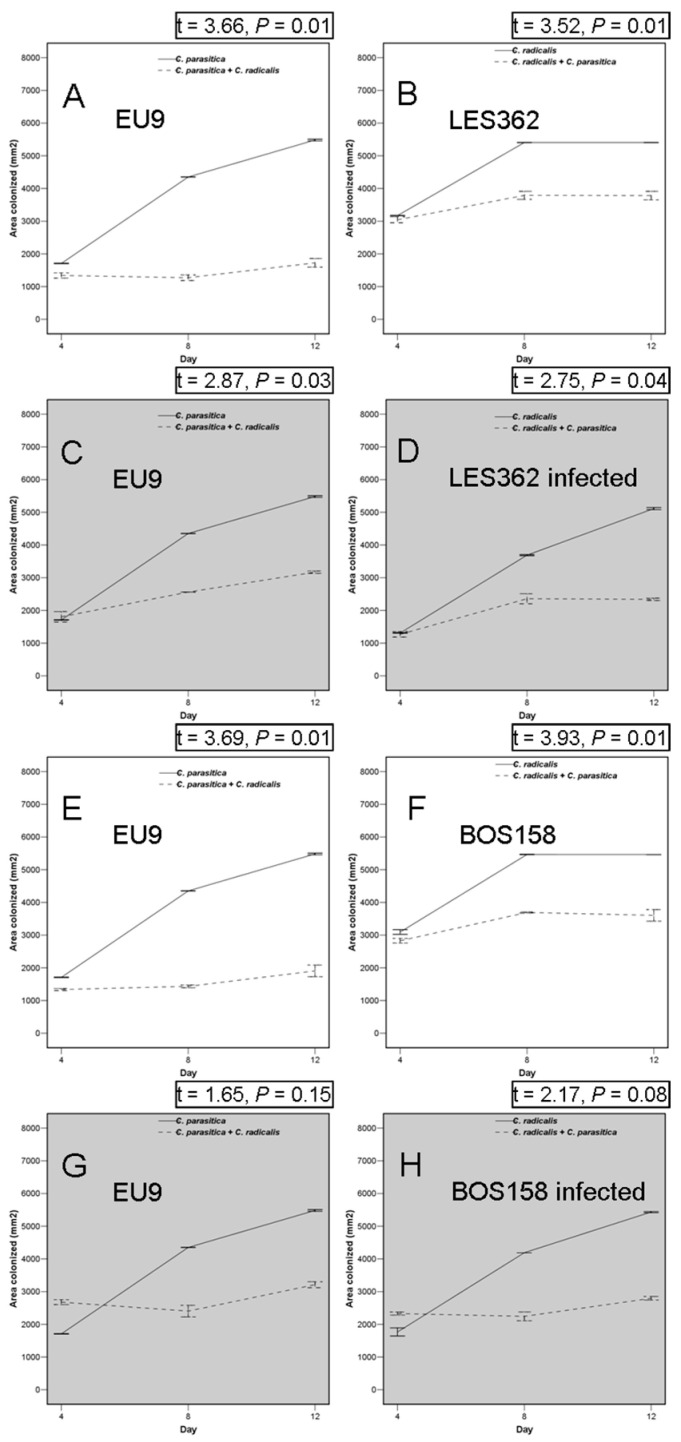
Primary resource capture capabilities of chestnut blight fungus, *Cryphonectria parasitica* EU9 vegetative compatibility group (**A**,**C**,**E**,**G**), interaction with *Cryphonectria radicalis* isolates LES362 and BOS158, non-infected or infected (highlighted in grey) by the CHV1 mycovirus (**B**,**D**,**F**,**H**). The significance of the decrease of each fungus growth areas in pairwise confrontation with the other fungus is indicated by the *p*-value of a paired-samples *t*-test. See Section 4.5 for experimental details. Continuous and dashed lanes respectively indicate the area colonised of each species growing in isolation or confronted with the other species.

The area of new substrate colonised by each fungus in pairwise combinations was always smaller than the area occupied by the same fungus grown in isolation (*t*-test always significant) (Figure 3), except in the case of virus-infected BOS158 for which there were no differences in *C. parasitica* (WAR706, VCG EU9) growing alone or in combination (Figure 3G, *t* = 1.65, *p* = 0.15) or BOS 158 growing alone or in combination (Figure 3H, *t* = 2.17, *p* = 0.08).

### 2.6. Secondary Capture

**Figure 4:**
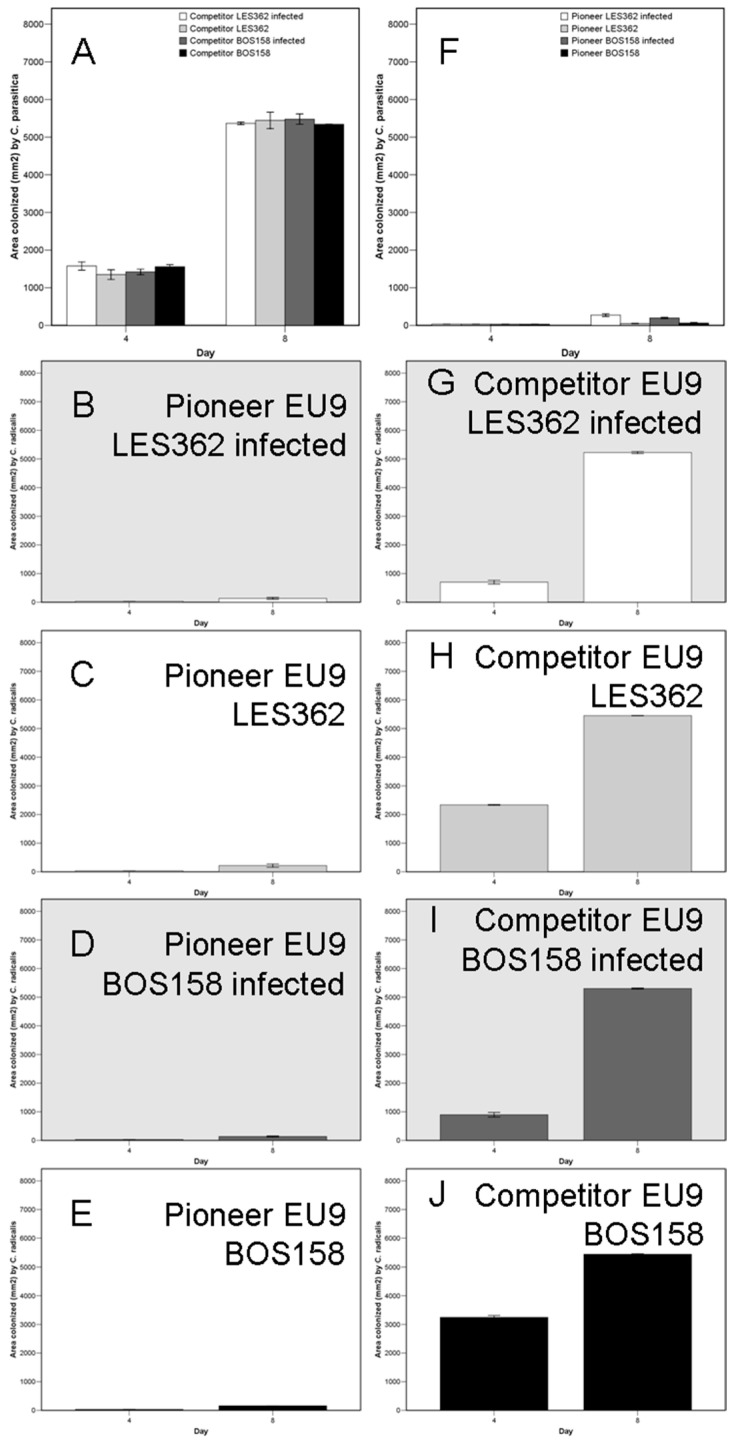
Secondary resource capture capabilities of chestnut blight fungus, *Cryphonectria parasitica* EU9 vegetative compatibility group (**A**,**F**), interaction with *Cryphonectria radicalis* isolates LES362 and BOS158, non-infected or infected (highlighted in grey) by the CHV1 mycovirus (**B**–**E**,**G**–**J**), when acting as a pioneer or a competitor. See Section 4.6 for experimental details.

When acting as a pioneer, *C. radicalis* almost totally outcompeted chestnut blight fungus (Figure 4F), more when it was virus-free (Figure 4G–J). However, when acting as a competitor, it was always almost totally overgrown by *C. parasitica* (Figure 4A–E).

### 2.7. Relative Virulence

**Figure 5:**
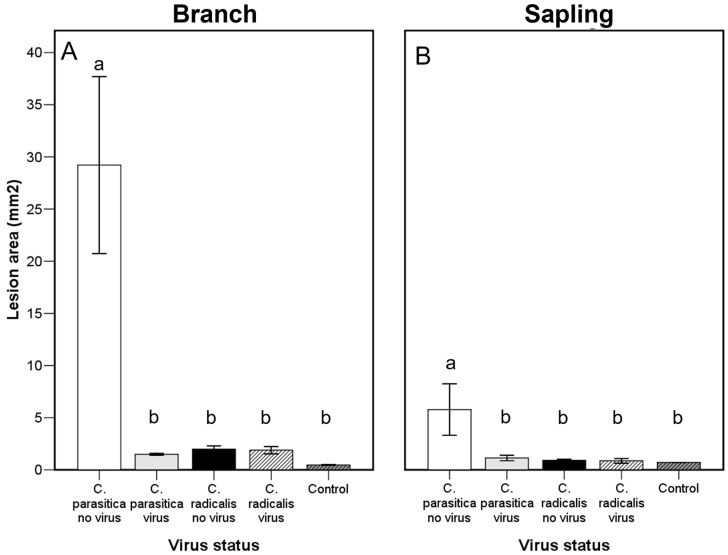
Lesion area (mean ± SE) produced by virus-infected and virus-free *C. parasitica* and *C. radicalis* or PDA control. Lettering indicates significant differences by treatment. (**A**) Branch segments; (**B**) saplings. See Section 4 last epigraph for experimental details.

At the end of the trial, all the branch segment material was dead (Table S1). The presence and number of epicormic shoots increased with the lesion area. The presence of perithecia increased with the presence of stromata. Perithecia presence was associated with certain *C. radicalis* isolates (ABB154, LES358, BOS157, BOS158). The lesion area of virus-free *C. parasitica* (WAR706, EU9) was significantly greater than all the other isolates tested. The lesion area of all *C. radicalis* isolates were not significantly different from the controls (Figure 5).

**Figure 6:**
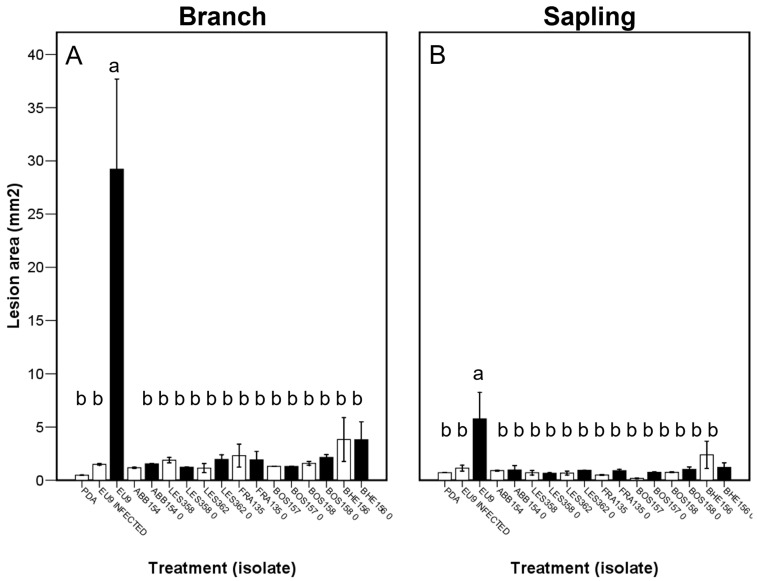
Lesion area (mean ± SE) produced by virus-infected (white bars) and virus-free (black bars) *C. parasitica* and *C. radicalis* or PDA control (white bar). Lettering indicates significant differences by treatment. (**A**) Branch segments; (**B**) saplings. See Section 4 last epigraph for experimental details.

Although all saplings were also dead by the end of the experiment, neither stromata nor perithecia were observed in the saplings (Table S1). A similar virulence pattern was observed to the branch segment trial, although virus-free *C. parasitica* lesion areas were smaller in the sapling trial (Figure 5). In a split-plot by isolate (Figure 6), there were no significant differences in virulence among any of the *C. radicalis* infected isolates and their respective virus-free controls, which provides further evidence that CHV1 infection does not induce a reduction of virulence in *C. radicalis*, in contrast with the effect it induces in *C. parasitica*.

### 2.8. Biocontrol Potential

**Figure 7:**
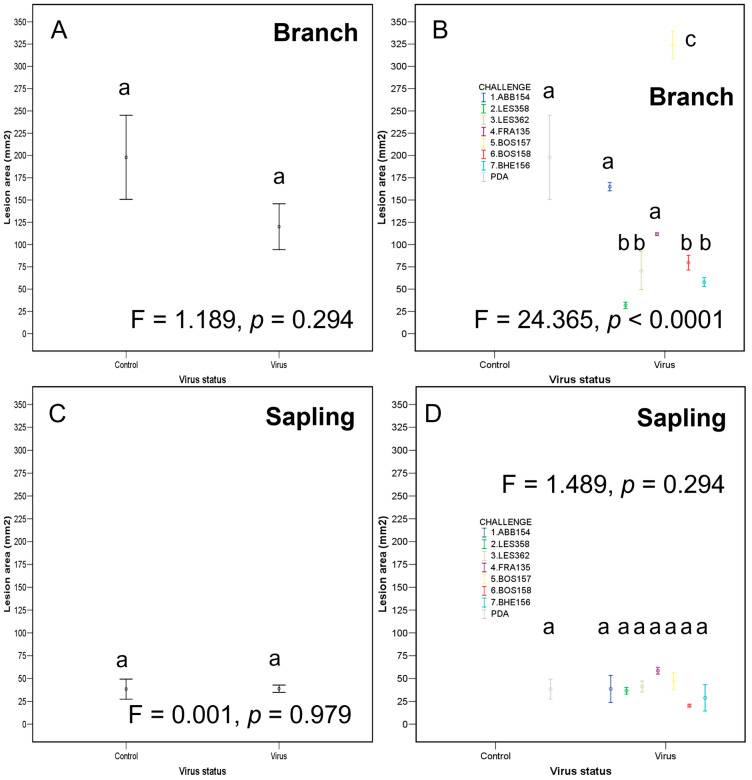
Lesion area (mean ± SE) by primary inoculation and challenge inoculation (assay II) with virus or without virus (control) using branches (**A**,**B**) or saplings (**C**,**D**). Different lettering indicates significant differences between the different challenge inoculations results. See Section 4 last epigraph for experimental details.

The results of Assay II are represented in Table S2 and Figure 7.

**Figure 8:**
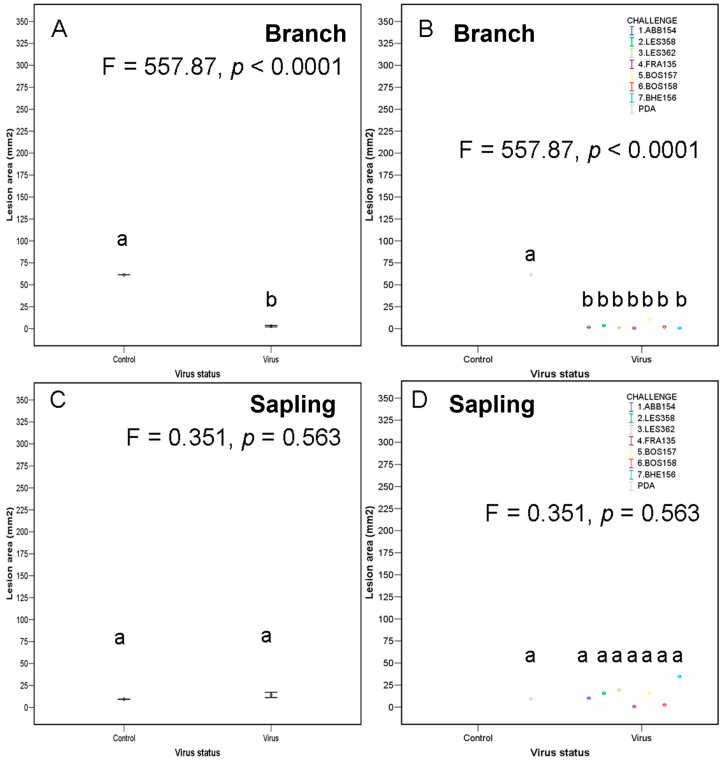
Lesion area (mean ± SE) by primary inoculation and challenge inoculation (assay II repeated, targeted) with virus or without virus (control) using branches (**A**,**B**) or saplings (**C**,**D**). Different lettering indicates significant differences between the different challenge inoculations results. See Section 4 last epigraph for experimental details.

Not all the branch segments were dead by the end of this experiment. Branch death frequency, and the presence of stromata increased with the lesion area (Table S2). In the challenge treatment using isolate BOS157, the lesion area was larger than those of the control (Figure 8), while four isolate treatments (LES358, LES362, BOS158 and BHE156) resulted in smaller lesions than the control.

Most, but not all saplings were dead at the end of the trial, but perithecia were never observed. Although virus concentration in *C. radicalis* was negatively correlated to the lesion area induced by *C. parasitica*, there were no significant differences in the lesion area observed between the virus-infected isolate treatments and the PDA control (Figure 7).

After repeating the same procedure but only using targeted VC-groups (see Table 4 for the respective selected combinations), the results are represented in Table S3 and Figure 8. In this case, the branch segments, after the same time, were still alive in some parts, and the saplings were chlorotic but predominantly alive. The branches treated with all virus-infected *C. radicalis* isolates had smaller lesion areas than the controls (Figure 8), but there were no significant differences when considering the saplings. There were several logical relationships found in the branch segments. For example, there were positive correlations between the lesion area and dead/alive status and the presence of stromata. It was also observed here that as the inoculum viral concentration increased, the more likely the branches were to be partially alive.

## 3. Discussion

Cryphonectria hypovirus 1 is known to induce hypovirulence in *C. parasitica* by reducing its growth, colour, sporulation and subsequent virulence when infecting both cut branches and saplings [8]. Therefore, the virus is used in Europe and some parts of the USA (inoculated in Virginia, Wisconsin, and Maryland) for chestnut blight control [14]. The virus has been introduced into continental Europe, and the UK (detected in England by Forest Research for the first time in November 2017), often associated with *C. parasitica* from countries in eastern Asia [17,18,19], known to be the geographical origin of the fungus. Those introductions are most likely to have occurred through trade in chestnut tree planting material within Europe and/or the importation of Asiatic planting stock often intended for use in resistance breeding programmes against the chestnut ink disease caused by *Phytophthora* x *cambivora* and *Phytophthora cinnamomi*. Six genetically distinct CHV1 subtypes have been identified in Europe (I, D, E, F1, F2 and G) [20,21]. Subtype I (also known as the Italian subtype), which is the only subtype that has been detected in England [6,7] (haplotype E-5 as used in the present study), is the most widespread due to its mild hypovirulence.

The VCG system in *C. parasitica* modulates CHV1 transmission by hyphal recognition and anastomosis, and thus determines the outcome of an epidemic [22]. It is regulated by six *vic* genes each with two possible alleles [23]. The VCG of a *C. parasitica* isolate causing canker on a tree must be determined before inoculating in the field with a hypovirulent isolate of a compatible VC group [14].

The main objective of this study was to determine the possibility and biological effects of transmitting CHV1 to *C. radicalis*, a possibility that was suspected to be feasible since its detection in two apparently naturally infected *C. radicalis* isolates in 2019 and 2020 in the London area [7]. In those two isolates, probably due to their low viral concentration (around 10–20 ng/µL only), there were no obvious phenotypic differences between CHV1-infected and isogenic CHV1-free isolates, gained by single spore culturing, of *C. radicalis* isolated from the wider environment. There is a certain level of CHV1 virus flow between the two species (*C. parasitica*–*C. radicalis*), like that of CHV1 between *C. parasitica* and *C. nitschkei*, sympatric in chestnut trees in East Asia [24].

However, after experimental interspecific cross-transfer to new recipient isolates (this study), CHV1 was quite easily transmitted vertically via around 77% asexual spores and horizontally to other strains of *C. radicalis* via hyphal contact and anastomosis.

CHV1-infected *C. radicalis* isolates exhibited a higher load of the virus in the younger colony parts (edges), reduced growth, and reduced colour phenotype, depending on virus concentration. Sectorial reduced growth has been shown in other fungi infected by different mycoviruses, e.g., *Beauveria bassiana* [25], *Colletotrichum fructicola* [26], *Cryphonectria carpinicola* [27], *Cryphonectria parasitica* [14], *Macrophomina phaseolina* [28], *Fusarium equiseti* [29]. To the best of our knowledge, this is the first successful transmission of CHV1 from *C. parasitica* to *C. radicalis* by co-cultivation [30]. In contrast, in previous transmissions to *C. radicalis* of other mycoviruses such as CnFGV1 (Cryphonectria naterciae FusagraVirus 1), the mycelium morphology and growth rate of virus-infected strains were unaffected compared to the virus-free isogenic strains [27].

The reverse transmission back to *C. parasitica* proved to be very difficult, and the in vitro and in planta results showed that infected *C. radicalis* isolates were unable to control cankers and are not suitable as biocontrol agents, at least when using chestnut saplings. In the cut branch assay, there were some promising results but until a proper characterisation method is developed of the vegetative incompatibility system in *C. radicalis*, we cannot conclude much more. Such future work would require sequence data generation from the different *C. radicalis vic* loci and the design of specific primers and sensitivity testing. Also, it would be interesting to determine the mating type system in *C. radicalis* because some isolates in the present study (directly producing perithecia during the plant trials) could be heterokaryons regarding mating types.

Future research should also focus on the secondary capture capabilities of *C. radicalis* when acting as a pioneer in chestnut plant material. It could potentially be used as a preventative treatment, for instance on neighbouring trees or in a woodland following removal of an infected tree, even if it cannot be used as a treatment for a tree if it is already infected.

Until then, the biocontrol potential of the CHV1-infected British isolates of *C. parasitica* was experimentally verified again and found to be dependent on the inoculum compatibility and on the virus concentration, both when using saplings and branches. The consistency of the plant material and the controlled conditions used in our assays gave us high confidence in our observations and the conclusions that could be drawn from the data in relation to the efficacy of those artificially (by previous co-culture [8]) infected English isolates. The unsuitability of CHV1-infected *C. radicalis* to act as an effective biocontrol agent against *C. parasitica* further highlights the need for exploration of the field biocontrol capabilities of hypovirulent *C. parasitica* isolates in the UK. This remains the only known biocontrol option with potential.

## 4. Materials and Methods

### 4.1. Sampling, Isolation and Preservation

Around 80 sites were intensively surveyed by the Forestry Commission from January 2017 to March 2024 in southern and mid-England (UK).

In the field, *C. sativa* trees were examined for the presence of typical blight symptoms. Edges of lesions were sampled by lifting small sections of the bark with a sharp chisel. Bark panels (5 × 8 cm^2^) were excised at the margin of each lesion. Bark samples were doubled-bagged and labelled with their Ordnance Survey (OS) grid coordinates.

In the laboratory, the panels were surface disinfected with 70% ethanol and isolations from the edge of lesions made onto 2% malt extract agar (MEA, 20 g/L malt extract, Oxoid, Basingstoke, UK) amended with 0.25 g/L of streptomycin sulphate salt (Sigma-Aldrich (St. Louis, MO, USA), (MA+S)). After 4 days at 20 °C in the dark, colonies were transferred to potato dextrose agar (PDA; 39 g/L potato dextrose, Oxoid) and incubated at 25 °C for 7 days before they were identified by ITS1-5.8S-ITS2 rDNA sequencing and sequences deposited in NCBI GenBank. Six plugs of each representative isolate were deposited in mapped triplicate cryotubes at the THDAS culture collection at −80 °C in 800 µL glycerol 22%, after first vortexing and flash-freezing the filled tubes through liquid nitrogen.

### 4.2. Direct and Reverse Transmissions of the CHV1 Virus

Initially, there was an attempt to transmit the CHV1 virus from the three highly infected *C. parasitica* isolates FTC687 (EU10, 415.95 ng/µL), WAR706 (EU9, 428.41 ng/µL) [8] and HYD574 (EU2, 588.52 ng/µL), gained indeed by past transmissions, into nine (ABB154, LES358, LES362, FRA135, BOS157, BOS158, BHE156, HAY113, and BOS159) isolates of *C. radicalis* (previously tested as negative for the virus). Mycelium plugs (0.5 cm in diameter) were taken from the centre of 7-day-old colonies of *C. parasitica* (donor) and *C. radicalis* (recipient) isolates. Then, the plugs of each isolate pair (recipient and donor) were placed 5 mm apart on a PDA in a 9 cm Petri dish, with three replicate pairings per plate forming a triangle. The plates were incubated for 7 days in the dark at 25 °C. After pairing, a toothpick method colony reverse-transcription PCR, without the needs of RNA isolation or electrophoresis, previously published by our team [8], was used. Viral concentration in the recipient was calculated.

One isolate (ABB154) tested positive for the CHV1 virus, confirmed by the absence of *C. parasitica* mycelium growing underneath by the real-time method described by Chandelier et al. (2019) [16], and the virus was further transmitted from ABB154 to six other *C. radicalis* isolates using the same methodology.

Then, from all the infected *C. radicalis* cultures arising from the previous transmission assays, an identical trial was implemented but, on this occasion, there was an attempt to transmit the mycovirus back to the seventy-four collection of virulent, virus-free *C. parasitica* isolates (EU1-74) European testers. Samples were processed as above.

### 4.3. Pigmentation, Spatial Differences in Viral Concentration Within Colony, Growth Rate, Sporulation Rate, and Vertical Transmission by Conidia

To determine if pigmentation changes may have occurred after virus infection and to discern whether the virus was more concentrated in the middle of the colonies, a 0.5 cm plug was taken with a flamed sterilised metallic borer from the centre and edge of the seven infected-*C. radicalis* isolates and isogenic non-infected controls to PDA plates (three replicates) and incubated at 25 °C for 7 days.

After this time, culture plate photographs were taken, colour (0—pink usual *C. radicalis* appearance; 1—intermediate; 2—white) were assigned to each culture, and the concentration of the CHV1 virus tested using the method described by Romon-Ochoa et al. [8].

For determining the growth rate, after those 7 days of incubation, colonies of *C. radicalis* were not completely circular, the colony areas were traced onto tracing paper and measured (in mm^2^) using the American Phytopathological Society Assess 2.0 program following manufacturer’s instructions.

To test the vertical transmission of the virus, single spore isolates were prepared by diluting 1:10 the product of vortexing a 0.5 cm mycelial plug (from the centre of each colony) into 100 µL of sterile distilled water. Sixty microliters were spread on new PDA with a sterile disposable spreader and incubated for 4 days under same conditions as above. After subculturing the five smallest and most discrete starting colonies onto new PDA, the single conidia colonies were tested for the virus after 7 days of incubation using the same molecular method [8].

Sporulation rate was obtained first by drawing an asterisk through the entire diameter of the plate with a scalpel in each colonised PDA plate, then adding 4 mL of Tween80 0.01%, and shaking at 180 rpm, at 20 °C, for 20 min in an orbital shaker (IncuShake MAXI, SciQuip, Newtown, Shropshire, UK). The concentration of conidia (conidia/mL) was calculated by using a Neubauer chamber hemocytometer together with a manual counter device.

### 4.4. In Vitro Differential Competition

For an assessment of competition in vitro, the two *C. radicalis* isolates with the highest (LES362) and lowest (BOS158) concentrations of the virus were selected, along with an isogenic uninfected control isolate per code. Competition between these isolates and the virus-free *C. parasitica* isolate WAR706 (VCG EU9) was assessed using de Wit replacement series [31,32]. In each pairwise combination, disks (0.5 cm in diameter) of colonised agar were aseptically removed from one-week-old actively growing colonies of each fungal isolates and randomly inoculated on plates of PDA at ratios of 0:1, 0.25:0.75, 0.5:0.5, 0.75:0.25, and 1:0 within a central grid of 4 cm × 5 cm (20 squares of 1 cm^2^ each) in a 9 cm diameter Petri dish. Each inoculum proportion treatment was replicated two times, and all plates were incubated at 25 °C in the dark. After 1 week, the areas occupied by each fungus were traced on transparent tracing paper and measured using APS Assess 2.0 program as indicated in Section 4.3. ANOVA was performed to test for deviations from linearity in the relationships between area colonised by each species and its inoculum proportion. Relative crowding coefficients (RCC) were calculated for each pairwise comparison. The RCC of species A to B was defined as ((area occupied by fungus A at 0.5:0.5)/(area occupied by fungus B at 0.5:0.5))/((area occupied by fungus A at 1:0)/(area occupied by fungus B at 1:0)). An RCC of 1 indicates equal competition, an RCC > 1 indicates that species A is outcompeting species B, and an RCC < 1 indicates that species B is outcompeting species A.

### 4.5. Primary Resource Capture

To quantify the ability of *C. parasitica* and virus-free and virus-infected *C. radicalis* (same isolates previously used) to colonise new substrate in pairwise combinations, one mycelial plug (0.5 cm in diameter) was taken from actively growing colonies of each fungus and placed face down on opposite sides of 9 cm Petri dishes of PDA (*n* = 2 plates for each pairwise combination). After 4 days at 25 °C in the dark and at 4 days intervals for 12 days thereafter, the area colonised by each fungus was measured as above and compared using a *t*-test comparison of paired-means with the mean area occupied by the same fungus grown in isolation.

### 4.6. Secondary Resource Capture

To quantify the ability of *C. parasitica* and virus-free and virus-infected *C. radicalis* to colonise a previously partially occupied substrate by other competing species, in a series of two opposite experiments per pairwise combination, one mycelial plug of the secondary competitor was placed one centimetre from the growing edge of a 4-days-old colony of the pioneer fungal species in each of two 9 cm Petri dishes (per combination) of PDA and incubated at 25 °C in the dark. Growing areas were recorded as above and added to the total at 4 days intervals for 12 days, and the mean colonised area by each fungus was analysed by ANOVA throughout the time of competition.

### 4.7. In Planta Experiments

Two types of bioassays (I, individual inoculations and II, challenge inoculations) were performed, each using two types of plant material: branch segments and saplings.

Sweet chestnut tree saplings (*C. sativa*) of approximately 1.5 m height and approximately 2 cm diameter at the root collar, were purchased from an English nursery free of the disease (Delamere, Plant Passport GB-S00/45) and grown in the nursery facility at Forest Research (Alice Holt) from May 2021 to May 2024. They were moved to a biosafety level 3 incubator (MLR-352-PE, PHCBI, Loughborough, Leicestershire, UK) at Forest Research’s Holt quarantine laboratory and acclimated to the incubator for a week with temperature-control (25/20 °C, day/night), circa 60% relative humidity, with a photoperiod of 16 h of light (3 bulbs on, 550 µmol/cm^2^).

Sweet chestnut branch segments (circa 2 cm diam.) were collected from a disease-free area (Haslemere) and cut into 25 cm lengths and stored for one week until use in a cold store (4 °C).

All plant material was provided with 10 mL tap water twice a week (on Mondays and Fridays). All inoculations were performed using mycelial plugs taken from actively growing cultures after seven days on PDA. The viral content in all isolates was re-checked at that point following the real-time procedure of Romon-Ochoa et al. [8].

For assay I (individual inoculations), the seven different virus-infected *C. radicalis* isolates, their isogenic virus-free controls plus *C. parasitica* EU9 (WAR706, virus-infected and virus-free) and PDA controls were inoculated. After inoculation, the holes (the inoculations were made by removing a bark disc (5 mm diam.) using a sterile cork borer) were sealed with LacBalsam (Compo, Eggenfelden, Germany). Two replicates were used. Thus, a total of 34 lesion areas per plant material type were measured after 45 days. At the time of harvesting, the length and width of the cankers, and their respective growth rate per day, were measured, and the canker area was calculated using the ellipse formula A = L/2 × W/2 × pi, where area equals half-length per half-width per pi number. For the branch segments, four branches were randomly held in buckets distributed across a map of 8.5 buckets (34 branches).

For assay II (challenge inoculations to estimate biocontrol potential), a total of 16 saplings and 16 branch segments were used. In the case of the branches, three branches were randomly placed in buckets distributed across a map of 5.3 buckets (16 branches). Two weeks following primary inoculations with virulent *C. parasitica* WAR706 (EU9), challenge inoculations were made with the seven virus-infected *C. radicalis* strains and PDA as a negative control. Eight inoculations, regularly distributed along the periphery of a virulent canker, were carried out. After secondary inoculation, the holes were sealed with sterile water-soaked sterile cotton, parafilm and aluminium foil. At the time of challenge, the length and width of the original cankers were measured. Canker expansion after the biocontrol treatments was measured after 45 days.

In all the assays, other phenotypic features were also recorded using a binary scoring system. These were the alive/chlorotic/dead status and the presence/absence of epicormic growth or callusing. The diameter of the stem at and above any callus (if present) was also measured with a calliper. At the end of the experiment, all lesions were sampled to verify virus content. Four bark samples (top, two from the centre, and bottom of the lesion) were taken using a biopsy needle (diameter 1.6 mm, Microlance 3, BD, Huesca, Spain), placed on MA+S, and incubated for 4 days at 20 °C. Mycelium was transferred onto PDA and incubated at 25 °C in the dark for 7 days, before being tested using the same real-time molecular method as above [8].

The whole procedure of assay II was repeated but only using targeted VCGs (see Table 4 for the respective selected combinations). Statistical analyses were conducted in SPSS (version 13.0). Analysis of Variance (ANOVA) (Type 2 F tests) was used to determine the significance of fixed effects. Estimated marginal means with pairwise contrasts (Tukey’s corrections) were used to show significant differences within fixed effects.

## 5. Conclusions

We characterised the biological features of the virus CHV1 artificially cross-infecting *Cryphonectria radicalis*. CHV1 is readily transmitted vertically via around 77% asexual spores and horizontally to other strains of *C. radicalis* via hyphal contact, while the reverse transmission back to *C. parasitica* proved to be very difficult. CHV1-infected *C. radicalis* isolates exhibited a higher viral load in the younger colony parts (edges), with reduced growth and reduced colour phenotype, all dependent on virus concentration. However, overall, several in vitro and in planta results showed that infected *C. radicalis* was unable to control cankers and is not suitable as biocontrol agent, at least until a characterisation method for the vegetative incompatibility system in *C. radicalis* is developed.

## Figures and Tables

**Table 1:** Isolates of the genus *Cryphonectria* used in this study.

#	Host	Collection ID	Year	CHV1 Detection (ng/µL)	Culture Preserved	Reference	County	GenBank Accession Number
	*C. parasitica*							
1	*Castanea sativa*	FTC687	2020	415.95	Yes	[8], this study	London	
2	*C. sativa*	WAR706	2021	428.41	Yes	[8], this study	Devon	
3	*C. sativa*	HYD574	2019	588.52	Yes	[8], this study	London	
	*C. radicalis*							
1	*C. sativa*	CHI71	2017		No	-	London	
2	*C. sativa*	HAY113	2017		No	-	Devon	
3	*C. sativa*	LES129a	2017		No	-	London	
4	*C. sativa*	BLA134	2017		No	-	London	
5	*C. sativa*	FRA135	2017		Yes	This study	London	PQ373832
6	*C. sativa*	BOS143	2017		No	-	London	
7	*C. sativa*	BOS144	2017		No	-	London	
8	*C. sativa*	ABB154	2017		Yes	This study	London	PQ373833
9	*C. sativa*	BHE156	2017		Yes	This study	London	PQ373834
10	*C. sativa*	BOS157	2017		Yes	This study	London	PQ373835
11	*C. sativa*	BOS158	2017		Yes	This study	London	PQ373836
12	*C. sativa*	BOS159	2017		Yes	-	London	
13	*C. sativa*	LES358	2018		Yes	This study	London	PQ373837
14	*C. sativa*	LES362	2018		Yes	This study	London	PQ373838
15	*C. sativa*	LES412	2018		No	-	London	
16	*C. sativa*	LES415	2018		No	-	London	
17	*C. sativa*	TAD429	2019		No	-	Surrey	
18	*C. sativa*	LES450	2019		No	-	London	
19	*C. sativa*	DEN498	2019		No	-	London	
20	*C. sativa*	JAC565	2019		No	-	London	
21	*C. sativa*	JAC566	2019	8.8	Yes	[7]	London	MT256128
22	*C. sativa*	KEN590	2020	19.6	Yes	[7]	London	MT256129
23	*C. sativa*	WCP609	2020		No	-	London	
24	*C. sativa*	ACO701	2021		No	-	Somerset	
25	*C. sativa*	ACO703	2021		No	-	Somerset	
26	*C. sativa*	KEN719	2021		No	-	London	
27	*C. sativa*	POWP733	2021		No	-	Devon	
28	*C. sativa*	POWP734	2021		No	-	Devon	
29	*C. sativa*	WATL739	2021		No	-	London	

**Table 2:** Cycle threshold (Ct) values and concentration (viral copies/µL) among crosses of three highly CHV1 virus infected *Cryphonectria parasitica* isolates against a collection of nine un-infected *Cryphonectria radicalis* cultures.

	Infected *C. parasitica* Isolate		
*C. radicalis*	FTC687 EU10	WAR706 EU9	HYD574 EU2
ABB154	- 0	24.33 333,833.11 copies/µL *	- 0
LES358	- 0	- 0	- 0
LES362	- 0	- 0	- 0
FRA135	- 0	- 0	- 0
BOS157	- 0	- 0	- 0
BOS158	- 0	- 0	- 0
BHE156	- 0	- 0	- 0
HAY113	- 0	- 0	- 0
BOS159	- 0	- 0	- 0

* See article [8] for copy number calculation regression equation.

**Table 3:** Cycle threshold (Ct) values and concentration (viral copies/µL) among crosses of the CHV1 virus infected *Cryphonectria radicalis* isolate ABB154 against the other six un-infected *Cryphonectria radicalis* cultures.

	Infected *C. radicalis*
*C. radicalis*	ABB154
LES358	25.30 173,075.38 *
LES362	23.94 434,745.69
FRA135	25.04 206,398.18
BOS157	23.54 570,009.84
BOS158	23.05 794,328.23
BHE156	24.41 316,227.76

* See article [8] for copy number calculation regression equation.

**Table 4:** Cycle threshold (Ct) values and concentration (viral copies/µL) among crosses of seven CHV1 virus infected-*Cryphonectria radicalis* isolates against the collection of EU1-74 vegetative compatibility groups of *Cryphonectria parasitica*.

			Infected *C. radicalis* Isolate				
*C. parasitica* VCG	ABB154	LES358	LES362	FRA135	BOS157	BOS158	BHE156
EU1							
EU2							
EU3							
EU4							
EU5							
EU6							
EU7							
EU8							
EU9						35 242.82 copies/µL *	
EU10							
EU11							
EU12							
EU13							
EU14							
EU15							
EU16	24.24 354,813.38 copies/µL	33.47 684.37 copies/µL	23.88 452,774.91 copies/µL	22.98 832,891.12 copies/µL	32.73 1129.64 copies/µL	24.7 259,839.92 copies/µL	34.6 318.37 copies/µL
EU17							
EU18							
EU19							
EU20			30.11 6660.84 copies/µL				
EU21							
EU22							
EU23							
EU24							
EU25							
EU26							
EU27							
EU28							
EU29							
EU30							
EU31							
EU32							
EU33							30.49 5149.49 copies/µL
EU34							
EU35							
EU36							
EU37							
EU38							
EU39							
EU40							
EU41							
EU42							
EU43							
EU44	29.24 12,006.37 copies/µL						
EU45							
EU46							
EU47							
EU48							
EU49		29.00 14,125.37 copies/µL					
EU50							
EU51							
EU52							
EU53							
EU54							
EU55							
EU56							
EU57							
EU58							
EU59							
EU60							
EU61							
EU62				26.97 55,854.58 copies/µL	27.09 51,494.95 copies/µL		
EU63							
EU64							
EU65							
EU66							
EU67							
EU68							
EU69							
EU70							
EU71							
EU72							
EU73							
EU74							

* See article [8] for copy number calculation regression equation.

**Table 5:** Colony growth rate (mm^2^) and colouration among the seven CHV1 virus-infected *C. radicalis* isolates (subbed from centre or edge), and isogenic control lanes.

*C. radicalis* Isolate	Type	Type Binary	Colour (0 Pink, 1 Intermediate, 2 White)	Growth Area (mm^2^)	Sporulation Rate (Conidia/µL)	Cycle Threshold (Ct) Value	CHV1 Virus Concentration (Copies/µL)
ABB154	Non-infected control	0	0	6361.72	710,000	- *	0
	Infected centre	1	2	4295.63	4,600,000	24.49	299,550.87
	Infected edge	2	2	2081.45	660,000	23.79	481,230.27
LES358	Non-infected control	0	0	4896.84	1,600,000	-	0
	Infected centre	1	2	4464.90	2,650,000	23.21	712,756.48
	Infected edge	2	1	3820.38	1,450,000	23.16	737,304.79
LES362	Non-infected control	0	0	6361.72	1,150,000	-	0
	Infected centre	1	2	2818.38	900,000	22.72	993,250.57
	Infected edge	2	2	2891.29	3,500,000	22.72	993,250.57
FRA135	Non-infected control	0	0	5202.92	1,820,000	-	0
	Infected centre	1	1	3320.24	1,650,000	25.36	166,183.62
	Infected edge	2	1	2104.34	2,550,000	16.71	58,170,913.29
BOS157	Non-infected control	0	0	6361.72	1,220,000	-	0
	Infected centre	1	1	4247.15	500,000	23.71	508,021.80
	Infected edge	2	2	2514.20	2,950,000	22.77	960,180.63
BOS158	Non-infected control	0	0	6361.72	1,220,000	-	0
	Infected centre	1	1	3313.50	4,600,000	23.26	689,025.49
	Infected edge	2	1	2320.32	2,300,000	23.68	518,448.81
BHE156	Non-infected control	0	0	6361.72	2,300,000	-	0
	Infected centre	1	1	3295.20	3,050,000	23.99	420,270.98
	Infected edge	2	1	2174.22	1,850,000	23.79	481,230.27
Pearson coefficient **		−0.830	−0.855	0.843	0.374	N/A	−0.049
*p*		0.0001	0.0001	0.0001	0.095	N/A	0.833

* See article [8] for copy number calculation regression equation. ** Correlation analysis against cycle threshold values. Significant correlations highlighted in grey.

**Table 6:** Cycle threshold (Ct) values and concentration (viral copies/µL) among the five smallest replicate single spore cultures of the seven CHV1 virus-infected *C. radicalis* isolates.

			Infected *C. radicalis* Isolate					
Single Spore Culture	ABB154	LES358	LES362	FRA135	BOS157	BOS158	BHE156	Mean
1	22.77 960,180.63 *	13.86 400,812,425.40	23.07 783,641.89	13.26 601,743,994.60	- 0	24.43 311,973.45	- 0	
2	23.90 446,683.59	23.67 521,971.82	25.43 158,489.31	- 0	23.27 684,374.97	24.35 399,341.95	- 0	
3	- -	25.75 127,609.30	17.09 44,971,893.81	27.92 29,352.63	28.28 23,001.95	27.71 33,838.55	26.35 84,998.59	
4	27.29 44,971.89	24.54 289,577.42	12.80 821,685,990.30	26.95 56,616.25	30.60 4779.82	- 0	- 0	
5	24.00 416,023.29	22.91 873,326.16	7.36 32,711,908,470.00	6.00 82,168,599,030.00	- 0	25.38 163,947.90	33.16 844.24	
% vertical transmission	80	100	100	80	60	80	40	77.14
Mean positives	24.49 299,550.87	22.14 1471,116.47	17.15 43,181,141.39	18.53 16,959,450.03	27.37 42,600.21	25.46 155,301.79	29.75 8499.85	23.55 566,162.59

* See article [8] for copy number calculation regression equation.

## Data Availability

The datasets generated and analysed during the current study are deposited in the FR THDAS repository and are available upon reasonable request.

## Supplementary Materials

The following supporting information can be downloaded at: https://www.mdpi.com/article/10.3390/ijms252212023/s1.
