# Supplementary material for: Transmission of Cryphonectria Hypovirus 1 (CHV1) to Cryphonectria radicalis and In Vitro and In Vivo Testing of Its Potential for Use as Biocontrol Against C. parasitica
Source: Int J Mol Sci. 2024 Nov 8;25(22):12023. doi: 10.3390/ijms252212023 (PMC11593397; doi:10.3390/ijms252212023)
Supplement: Supplementary file 1 [file ijms-25-12023-s001.zip › ijms-3286971-supplementary.pdf]

Supplementary Materials

Table S1. Correlation results among all tested parameters in assay I, respectively using branches and saplings.

**ASSAY I, BRANCHES**		**Lesion area**	**Inoculum concentration**	**Final concentration**	**Alive (0), Dead (1)**	**Epicormics no (0), yes (1)**	**Epicormics shoots number**	**Fruit bodies no (0), yes (1)**	**Perithecia no (0), yes (1)**
Lesion area	Pearson correlation	1							
	Sig. (2-tailed)								
Inoculum concentration	Pearson correlation	-0.099	1						
	Sig. (2-tailed)	0.579							
Final concentration	Pearson correlation	-0.098	0.998	1					
	Sig. (2-tailed)	0.580	0.0001						
Alive (0), Dead (1)	Pearson correlation	-*	-*	-*	1				
	Sig. (2-tailed)	-	-	-					
Epicormics no (0), yes (1)	Pearson correlation	0.870	-0.062	-0.062	-*	1			
	Sig. (2-tailed)	0.0001	0.726	0.726	-				
Epicormics shoots number	Pearson correlation	0.870	-0.062	-0.062	-*	1.000	1		
	Sig. (2-tailed)	0.0001	0.726	0.726	-	0.0001			
Fruiting bodies no (0), yes (1)	Pearson correlation	-0.164	0.073	0.075	-*	-0.120	-0.120	1	
	Sig. (2-tailed)	0.353	0.680	0.673	-	0.498	0.498		
Perithecia no (0), yes (1)	Pearson correlation	0.207	0.282	0.283	-*	-0.164	-0.164	0.482	1
	Sig. (2-tailed)	0.240	0.107	0.105	-	0.354	0.354	0.004	
Treatment (isolate) number	Pearson correlation	-0.211	0.127	0.138	-*	-0.216	-0.216	0.267	0.411
	Sig. (2-tailed)	0.230	0.474	0.435	-	0.220	0.220	0.127	0.016
**ASSAY I, SAPLINGS**		**Lesion area**	**Inoculum concentration**	**Final concentration**	**Alive (0), Dead (1)**	**Epicormics no (0), yes (1)**	**Epicormics shoots number**	**Fruit bodies no (0), yes (1)**	**Perithecia no (0), yes (1)**
Lesion area	Pearson correlation	1							
	Sig. (2-tailed)								
Inoculum concentration	Pearson correlation	-0.182	1						
	Sig. (2-tailed)	0.304							
Final concentration	Pearson correlation	-0.184	0.999	1					
	Sig. (2-tailed)	0.298	0.0001						
Alive (0), Dead (1)	Pearson correlation	-*	-*	-*	1				
	Sig. (2-tailed)	-	-	-					
Epicormics no (0), yes (1)	Pearson correlation	-0.033	-0.198	-0.193	-*	1			
	Sig. (2-tailed)	0.243	0.261	0.274	-				
Epicormics shoots number	Pearson correlation	-0.031	-0.199	-0.193	-*	0.912	1		
	Sig. (2-tailed)	0.862	0.260	0.274	-	0.0001			
Fruiting bodies no (0), yes (1)	Pearson correlation	-*	-*	-*	-*	-*	-*	1	
	Sig. (2-tailed)	-	-	-	-	-	-		
Perithecia no (0), yes (1)	Pearson correlation	-*	-*	-*	-*	-*	-*	-*	1
	Sig. (2-tailed)	-	-	-	-	-	-	-	
Treatment (isolate) number	Pearson correlation	-0.172	0.127	0.141	-*	0.301	0.415	-*	-*
	Sig. (2-tailed)	0.331	0.474	0.425	-	0.084	0.015	-	-

(dark grey)	Negative correlation is significant at the 0.05 level.
(light grey)	Positive correlation is significant at the 0.05 level.

* Cannot be computed because at least one of the variables is constant.

Table S2. Correlation results among all tested parameters in assay number II, respectively using branches and saplings.

**ASSAY II, BRANCHES**		**Lesion area**	**Inoculum concentration**	**Final concentration**	**Alive (0), Dead (1)**	**Epicormics no (0), yes (1)**	**Epicormics shoots number**	**Fruit bodies no (0), yes (1)**	**Perithecia no (0), yes (1)**
Lesion area	Pearson correlation	1							
	Sig. (2-tailed)								
Inoculum concentration	Pearson correlation	-0.132	1						
	Sig. (2-tailed)	0.626							
Final concentration	Pearson correlation	-0.132	1.000	1					
	Sig. (2-tailed)	0.626	0.0001						
Alive (0), Dead (1)	Pearson correlation	0.823	-0.269	-0.269	1				
	Sig. (2-tailed)	0.0001	0.314	0.314					
Epicormics no (0), yes (1)	Pearson correlation	-*	-*	-*	-*	1			
	Sig. (2-tailed)	-	-	-	-				
Epicormics shoots number	Pearson correlation	-*	-*	-*	-*	-*	1		
	Sig. (2-tailed)	-	-	-	-	-			
Fruiting bodies no (0), yes (1)	Pearson correlation	0.616	0.410	0.410	0.656	-*	-*	1	
	Sig. (2-tailed)	0.011	0.115	0.115	0.006	-	-		
Perithecia no (0), yes (1)	Pearson correlation	0.379	-0.239	-0.239	0.234	-*	-*	-0.051	1
	Sig. (2-tailed)	0.147	0.373	0.373	0.384	-	-	0.851	
Treatment (donor isolate)	Pearson correlation	-0.145	0.056	0.056	-0.252	-*	-*	-0.192	0.088
	Sig. (2-tailed)	0.592	0.836	0.836	0.346	-	-	0.475	0.745
**ASSAY II, SAPLINGS**		**Lesion area**	**Inoculum concentration**	**Final concentration**	**Alive (0), Dead (1)**	**Epicormics no (0), yes (1)**	**Epicormics shoots number**	**Fruit bodies no (0), yes (1)**	**Perithecia no (0), yes (1)**
Lesion area	Pearson correlation	1							
	Sig. (2-tailed)								
Inoculum concentration	Pearson correlation	-0.550	1						
	Sig. (2-tailed)	0.027							
Final concentration	Pearson correlation	-0.550	1.000	1					
	Sig. (2-tailed)	0.027	0.0001						
Alive (0), Dead (1)	Pearson correlation	-*	-*	-*	1				
	Sig. (2-tailed)	-	-	-					
Epicormics no (0), yes (1)	Pearson correlation	0.405	0.013	0.013	-*	1			
	Sig. (2-tailed)	0.120	0.963	0.963	-				
Epicormics shoots number	Pearson correlation	0.446	-0.202	-0.202	-*		1		
	Sig. (2-tailed)	0.083	0.452	0.452	-				
Fruiting bodies no (0), yes (1)	Pearson correlation	-0.009	0.148	0.148	-*	-0.051	-0.217	1	
	Sig. (2-tailed)	0.973	0.584	0.584	-	0.851	0.419		
Perithecia no (0), yes (1)	Pearson correlation	-*	-*	-*	-*	-*	-*	-*	1
	Sig. (2-tailed)	-	-	-	-	-	-	-	
Treatment (donor isolate)	Pearson correlation	-0.211	0.056	0.056	-*	0.027	-0.086	0.088	-*
	Sig. (2-tailed)	0.433	0.836	0.836	-	0.919	0.753	0.745	-

(dark grey)	Negative correlation is significant at the 0.05 level.
(light grey)	Positive correlation is significant at the 0.05 level.

* Cannot be computed because at least one of the variables is constant.

Table S3. Correlation results among all tested parameters in repeated assay II, using also branches and saplings, targeting certain *C. parasitica* VC-groups.

**ASSAY II TARGETED, BRANCHES**		**Lesion area**	**Inoculum concentration**	**Final concentration**	**Alive (0), Dead (1)**	**Epicormics no (0), yes (1)**	**Epicormics shoots number**	**Fruit bodies no (0), yes (1)**	**Perithecia no (0), yes (1)**
Lesion area	Pearson correlation	1							
	Sig. (2-tailed)								
Inoculum concentration	Pearson correlation	-0.205	1						
	Sig. (2-tailed)	0.447							
Final concentration	Pearson correlation	-0.281	0.912	1					
	Sig. (2-tailed)	0.292	0.0001						
Alive (0), Dead (1)	Pearson correlation	0.581	-0.561	0.419	1				
	Sig. (2-tailed)	0.018	0.024	0.107					
Epicormics no (0), yes (1)	Pearson correlation	-*	-*	-*	-*	1			
	Sig. (2-tailed)	-	-	-	-				
Epicormics shoots number	Pearson correlation	-*	-*	-*	-*	-*	1		
	Sig. (2-tailed)	-	-	-	-	-			
Fruiting bodies no (0), yes (1)	Pearson correlation	0.554	0.687	0.566	0.856	-*	-*	1	
	Sig. (2-tailed)	0.026	0.003	0.022	0.0001	-	-		
Perithecia no (0), yes (1)	Pearson correlation	-0.305	0.639	0.484	0.302	-*	-*	-*	1
	Sig. (2-tailed)	0.251	0.008	0.058	0.256	-	-	-	
Treatment (donor isolate)	Pearson correlation	-0.560	0306	0.273	-0.132	-*	-*	-0.169	0.327
	Sig. (2-tailed)	0.024	0.249	0.307	0.627	-	-	0.531	0.216
**ASSAY II TARGETED, SAPLINGS**		**Lesion area**	**Inoculum concentration**	**Final concentration**	**Alive (0), Dead (1)**	**Epicormics no (0), yes (1)**	**Epicormics shoots number**	**Fruit bodies no (0), yes (1)**	**Perithecia no (0), yes (1)**
Lesion area	Pearson correlation	1							
	Sig. (2-tailed)								
Inoculum concentration	Pearson correlation	1							
	Sig. (2-tailed)								
Final concentration	Pearson correlation	-0.438	1						
	Sig. (2-tailed)	0.090							
Alive (0), Dead (1)	Pearson correlation	-0.347	0.912	1					
	Sig. (2-tailed)	0.188	0.0001						
Epicormics no (0), yes (1)	Pearson correlation	0.627	-0.133	0.025	1				
	Sig. (2-tailed)	0.009	0.623	0.928					
Epicormics shoots number	Pearson correlation	0.051	-0.223	-0.132	-*	1			
	Sig. (2-tailed)	0.852	0.406	0.627	-				
Fruiting bodies no (0), yes (1)	Pearson correlation	0.499	-0.221	-0.056	-0.577	-*	1		
	Sig. (2-tailed)	0.049	0.411	0.837	0.019	-			
Perithecia no (0), yes (1)	Pearson correlation	-*	-*	-*	-*	-*	-*	1	
	Sig. (2-tailed)	-	-	-	-	-	-		
Treatment (donor isolate)	Pearson correlation	0.326	0.306	0.273	0.109	0.252	0.601	-*	1
	Sig. (2-tailed)	0.218	0.249	0.307	0.688	0.346	0.014	-	

(dark grey)	Negative correlation is significant at the 0.05 level.
(light grey)	Positive correlation is significant at the 0.05 level.
